# Successful Use of Pulmonary Vasodilators in Acute Chest Syndrome Complicated by Persistent Right Ventricular Failure

**DOI:** 10.1155/2019/4681392

**Published:** 2019-04-18

**Authors:** Jacqueline T. DesJardin, Lucas S. Zier

**Affiliations:** ^1^Department of Internal Medicine, University of California San Francisco, San Francisco, CA, USA; ^2^Division of Cardiology, Zuckerberg San Francisco General Hospital and Trauma Center, San Francisco, CA, USA

## Abstract

Pulmonary hypertension (PH) is a known consequence of sickle cell disease (SCD) and is associated with increased mortality and more frequent episodes of acute chest syndrome (ACS). Pulmonary pressures are known to increase during ACS, and right ventricular (RV) failure has been described as a significant cause of mortality in this condition. Management of ACS includes exchange transfusion, pain control, and prevention of hypovolemia and hypoxemia. However, in patients with a history of precapillary PH in whom ACS is complicated by persistent RV failure and cardiogenic shock, RV afterload reduction with pulmonary vasodilators may be an effective treatment strategy. Here, we present a case of a young man with SCD-associated PH and ACS who was successfully managed with inhaled and oral pulmonary vasodilators in the setting of persistent elevations in pulmonary vascular resistance leading to acute RV failure and cardiogenic shock.

## 1. Introduction

Pulmonary hypertension (PH) occurs in 10% of patients with sickle cell disease (SCD) and can be either pre- or postcapillary [1]. Due to anemia and reduced blood viscosity in SCD, even mild elevations in pulmonary vascular resistance (PVR) are indicative of precapillary physiology [2]. Precapillary SCD-associated PH is thought to be caused by reduced bioavailability of nitric oxide during hemolysis, chronic thromboembolism, and chronic hypoxia [3]. Six-year mortality in SCD-associated PH is 37%, and those with a higher transpulmonary gradient and PVR have worse outcomes [4, 5]. There may be a relationship between PH and risk of acute chest syndrome (ACS) given elevated pulmonary artery pressures (PAPs) have been associated with more frequent episodes of ACS [6].

The pathophysiology of ACS is complex and poorly understood; however, fundamentally ACS is due to vasoocclusion and/or vasoconstriction of the pulmonary microvasculature. PAPs increase during vasoocclusive pain crises and ACS, leading to increased RV afterload and the potential for RV failure [7, 8]. In a study of 70 patients admitted to the intensive care unit with ACS, acute RV failure occurred in 11 patients (16%) and was associated with a 40% mortality due to cardiogenic shock and multiple organ failure [8]. RV failure in ACS is often due to acute pressure overload in patients with baseline PH and RV dysfunction [9]. However, treatment of persistent RV failure in ACS is not well understood, particularly when PVR remains elevated after reversible causes of ACS have been addressed. Pulmonary vasodilators have not been formally studied in this setting, although they may have a role given our current physiologic understanding of ACS and RV failure.

Here, we present the case of a man with SCD complicated by PH who developed persistent RV failure and cardiogenic shock after an episode of ACS and was successfully managed with pulmonary vasodilators.

## 2. Case Presentation

A 38-year-old man with homozygous SCD presented to the emergency department with a sickle cell vasoocclusive pain crisis. On presentation, he reported one day of worsening midsternal chest pain and lower back pain. His initial workup was notable for normal vital signs, hemoglobin of 10.7 g/dL (baseline: 12.0 g/dL), absolute reticulocyte count of 374 k/*μ*L, creatinine of 1.16 mg/dL, lactic acid of 1.5 mmol/L, undetectable troponin, and initial chest radiograph with clear lungs.

**Figure 1:**
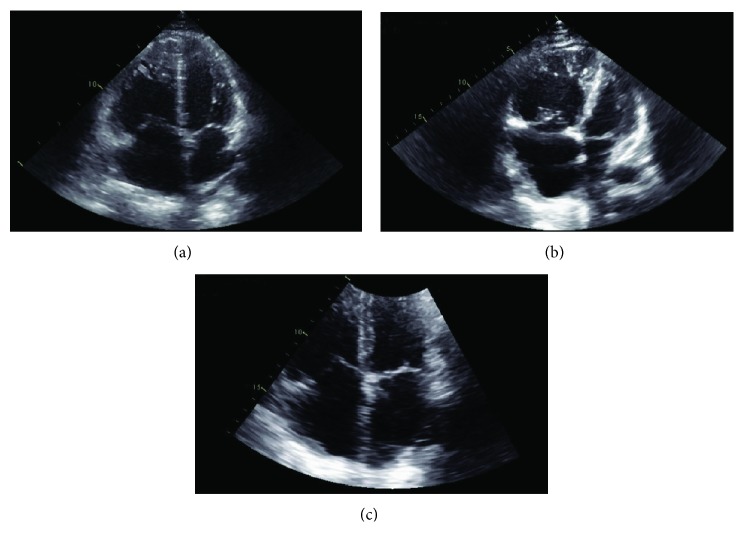
Apical four-chamber views and abbreviated echocardiogram reports from the baseline, acute chest syndrome (ACS), and follow-up. (a) Baseline echocardiogram. Twenty-two months prior to ACS. Left ventricular ejection fraction 55-60%. Moderately enlarged right ventricular chamber with mildly reduced systolic function. Mild right atrial enlargement. Moderate tricuspid regurgitation. Pulmonary artery systolic pressure 60 mmHg plus central venous pressure. Inferior vena cava small and collapses with inspiration. (b) Echocardiogram during ACS. Left ventricular ejection fraction 60-65%. Severely enlarged right ventricular chamber with moderately reduced systolic function. Severe right atrial enlargement. Severe tricuspid regurgitation. Pulmonary artery systolic pressure 132 mmHg plus central venous pressure. Inferior vena cava dilated. (c) Follow-up echocardiogram. Ten months after ACS. Left ventricular ejection fraction 55-60%. Moderately enlarged right ventricular chamber with moderately reduced systolic function. Moderate right atrial enlargement. Moderate tricuspid regurgitation. Pulmonary artery systolic pressure 45 mmHg plus central venous pressure. Inferior vena cava normal in caliber and collapses with inspiration.

On further chart review, he had a history of remote pulmonary embolism and was on life-long warfarin therapy and a history of precapillary PH. Echocardiogram twenty-two months prior to admission showed a moderately enlarged RV with mildly reduced function and estimated pulmonary artery systolic pressure (ePASP) of 60 mmHg (Figure 1(a)). Right heart catheterization (RHC) nineteen months prior to admission showed mild precapillary PH (Table 1). Pulmonary function testing showed forced expiratory volume in one second (FEV1) 72% predicted, forced vital capacity (FVC) 83% predicted, FEV1/FVC 71% predicted, and corrected diffusion capacity 45% predicted. A sleep study showed nocturnal desaturations and an apnea hypopnea index of 9.5, and he was placed on continuous positive airway pressure with supplemental oxygen at night. His PH was felt to be multifactorial given his history of SCD, mild obstructive sleep apnea and nocturnal hypoxia, and prior pulmonary embolism.

**Figure 2:**
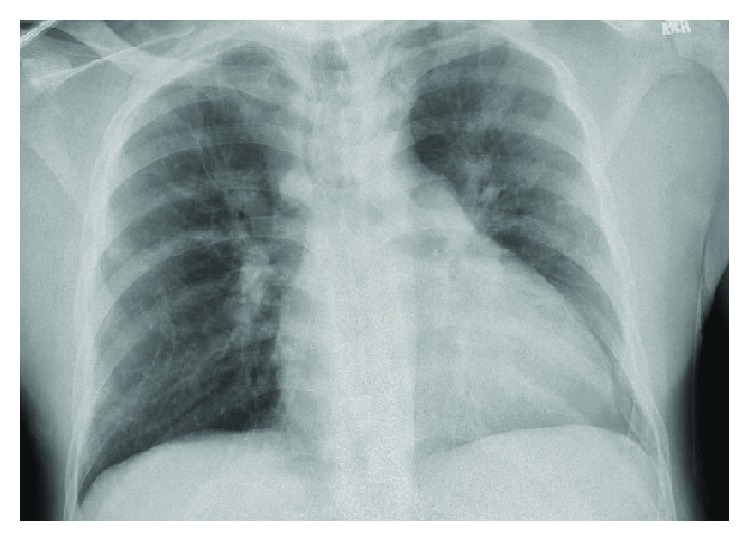
Chest radiograph from time of acute chest syndrome diagnosis.

Following admission, he was started on intravenous fluids and opiates. On hospital day two, his oxygen saturation dropped to 83% and blood pressure to 76/55 mmHg. Arterial blood gas on 6 L/min of oxygen via nasal cannula showed a partial pressure of arterial oxygen (PaO_2_) of 65 mmHg. He was placed on high-flow nasal cannula at 40 L/min and 50% fraction of inspired oxygen with improvement in PaO_2_ to 105 mmHg. Laboratory workup was notable for hemoglobin 8.5 g/dL, reticulocyte count 349 k/*μ*L, 55.2% hemoglobin S, lactic acid 5.0 mmol/L, lactate dehydrogenase 528 U/L, total bilirubin 1.4 mg/dL, creatinine 1.41 mg/dL, brain natriuretic peptide 586 pg/mL, and troponin 4.09 ng/mL. Chest radiograph showed slight left greater than right suprahilar opacities (Figure 1(b)), and computed tomography angiogram did not demonstrate a pulmonary embolism. Echocardiogram revealed a severely enlarged RV with severely reduced systolic function and an ePASP of 132 mmHg (Figure 2). There was concern for ACS. The patient received empiric vancomycin, meropenem, and azithromycin and underwent exchange transfusion of three units packed red blood cells with improvement in hemoglobin S to 26%. Despite these therapies, he had worsening hypotension over the following twenty-four hours, including requiring up to three vasopressors (norepinephrine, vasopressin, and epinephrine). Cardiology was consulted and, given the unclear etiology of his decompensation, a RHC with a retained pulmonary artery catheter was rapidly performed and demonstrated severely decompensated precapillary PH, acute RV failure, and cardiogenic shock (Table 1).

He was diuresed and started on dobutamine. Given his predominantly precapillary PH on RHC, he was also initiated on inhaled epoprostenol to allow for RV afterload reduction and avoid intubation (which was considered extremely high risk). He initially showed rapid improvement and was weaned from epinephrine and vasopressin in the following twelve hours. However, he failed to improve further despite effective diuresis, normal oxygenation, empiric antibiotics, and additional exchange transfusions. He continued to require high-dose dobutamine and inhaled epoprostenol for the following four days. Cardiac index, calculated by the indirect Fick method using a central venous catheter, remained 1.7-1.9 L/min/m^2^, and attempts to wean inotropic support resulted in hypotension. After several days of inability to wean dobutamine, it became clear that he was in persistent RV failure. Alternative therapies were considered to decrease RV afterload and allow for downtitration of inotropic and inhaled vasodilator support.

Phosphodiesterase inhibitors were avoided due to evidence of increased adverse effects in patients with SCD [7]. Endothelin receptor antagonists were avoided because of their potential to cause anemia and fluid retention, especially given the patient's low hemoglobin, active hemolysis, and decompensated heart failure. Intravenous therapies were considered, although they are not available at our institution and transfer to a tertiary center was declined.

Given that the patient had responded well to inhaled epoprostenol, it was felt that an oral agent acting upon the prostanoid pathway was appropriate to trial. We reviewed the literature and found that rapid uptitration of selexipag had been safe in healthy subjects and patients with PH [10–13]. Ultimately, he was initiated on rapidly uptitrated doses of selexipag, reaching target dosing of 1600 *μ*g twice daily over fifteen days. During the rapid uptitration, the patient denied symptoms associated with prostacyclin intolerance. Two days after initiation of selexipag, cardiac index improved to greater than 2 L/min/m^2^, and right atrial pressure decreased to less than 10 mmHg; eight days after initiation, he was able to be weaned completely from dobutamine and epoprostenol. He was discharged on hospital day 21 and followed up in the cardiology clinic twice in the following year without further episodes of ACS or RV failure. Follow-up echocardiogram ten months after hospitalization on selexipag and macitentan showed improved RV size and function with ePASP 45 mmHg (Figure 1(c)).

## 3. Discussion

The mainstay of therapy for ACS is adequate and immediate pain control, prevention of hypovolemia and hypoxemia, empiric antibiotics, and exchange transfusion to achieve hemoglobin S < 30%. ACS can be caused by infection, pulmonary embolization, and asthma exacerbation, or the trigger can be unknown. However, in patients with a history of PH, ACS may present with worsening RV failure. Acute RV failure from decompensated pulmonary arterial hypertension (PAH) is treated with pulmonary vasodilators, inotropic agents, and preload optimization. However, in treating this patient, we learned that little evidence exists to guide management of ACS in SCD when precapillary PH and RV failure persists following the resolution of reversible triggers.

Use of pulmonary vasodilators in SCD-associated PH has been understudied in general, not just in ACS. A randomized controlled trial comparing bosentan to placebo in SCD-associated PH was terminated early due to underenrollment [14]. The walk-PHaSST trial was stopped early due to increased pain crises in patients receiving sildenafil; furthermore, therapy with sildenafil did not improve hemodynamics or exercise capacity [7]. The American Thoracic Society recommends a trial of either a prostacyclin agonist or an endothelin receptor antagonist in patients with SCD and precapillary PH [2]. However, these recommendations apply to stable outpatients, and the efficacy of vasodilators in ACS is largely unknown.

Inhaled nitric oxide is the best-studied pulmonary vasodilator in treatment of vasoocclusive crises and ACS. In vasoocclusive pain crises, small trials have suggested that iNO is beneficial [15, 16], although this was not confirmed in a larger multicenter trial [17]. In ACS, several case reports have suggested that iNO is safe and effective [18, 19]. However, the only randomized placebo-controlled trial of iNO in ACS did not show statistically significant differences in mortality or need for endotracheal intubation. Notably, patients with hypotension were excluded from this trial [20]. It may be the case that pulmonary vasodilators are beneficial in subgroups of patients who experience RV failure and refractory hypotension as a predominant component of ACS. Other medications, such as epoprostenol or oral pulmonary vasodilators, have not been studied in ACS. Nonetheless, intravenous pulmonary vasodilators and/or iNO are standard of care in decompensated PAH [21].

In this case, we chose to initiate pulmonary vasodilators only when RV failure persisted after the acute ACS event; all other parameters (oxygenation, diuresis, and inotropy) had been optimized, and invasive hemodynamics confirmed severely decompensated precapillary PH. We used inhaled epoprostenol and, later, selexipag for RV afterload reduction because of limited access to intravenous prostanoid therapies. It is difficult to determine if the clinical improvement seen in this patient was due to pulmonary vasodilator therapy or delayed resolution of ACS; however, hemodynamic improvement occurred only after vasodilator initiation. Improved afterload reduction with pulmonary vasodilators may have allowed for more successful downtitration of inotropic therapy. In our interpretation, this case demonstrates the potential utility of RV afterload reduction with pulmonary vasodilators in nontraditional clinical settings such as ACS. Certainly, definitive management conclusions cannot be drawn from a single case report. However, given what is known about the pathophysiology of ACS, SCD-associated PH, and RV failure, pulmonary vasodilation should be studied further among patients in whom ACS is complicated by decompensated precapillary PH and cardiogenic shock.

## Figures and Tables

**Table 1:** Hemodynamics on right heart catheterization nineteen months prior to hospitalization (baseline) in comparison to during acute chest syndrome (ACS).

	Baseline	ACS
Mean right atrial pressure	5 mmHg	18 mmHg
Right ventricular pressure	40/3 mmHg	89/10 mmHg
Pulmonary artery pressure (mean)	42/16 (25) mmHg	95/46 (58) mmHg
Pulmonary capillary wedge pressure	6 mmHg	10 mmHg
Pulmonary vascular resistance	3.9 Wood units	13.6 Wood units
Cardiac output by thermodilution	4.83 L/min	3.53 L/min
Cardiac index by thermodilution	2.57 L/min/m^2^	1.86 L/min/m^2^
Arterial oxygen saturation	97%	98%
Pulmonary artery oxygen saturation	69%	53%
Hemoglobin	12.4 g/dL	10.6 g/dL
